# HIV Testing in Patients Diagnosed With CIN2 or Worse: Experience at The Jessop Wing Colonoscopy Unit, Sheffield, UK

**DOI:** 10.1155/ogi/5552384

**Published:** 2026-06-23

**Authors:** Victoria L. Parker, Ella Hodson, Rachel E. Lyon, Benjamin I. Stone, Madeleine C. Macdonald, Julia E. Palmer

**Affiliations:** ^1^ Department of Gynaecological Oncology, Sheffield Teaching Hospitals NHS Foundation Trust, Sheffield, UK, nhs.uk; ^2^ Division of Clinical Medicine, The University of Sheffield, Sheffield, UK, sheffield.ac.uk; ^3^ Department of Infectious Diseases, Sheffield Teaching Hospitals NHS Foundation Trust, Sheffield, UK, nhs.uk

**Keywords:** cervical screening, CIN2, colposcopy, high-grade dyskaryosis, HIV testing

## Abstract

**Background and aims:**

Debate regarding the value of HIV screening in English colposcopy services is ongoing. With no national guidance provided by the NHSCSP, few colposcopy services offer HIV testing. Sheffield colposcopy introduced standardised HIV testing in January 2023. We assessed prevalence rates and acceptability to HIV testing in our referred population with moderate or worse dyskaryosis, CIN2 or worse.

**Methods:**

A retrospective service evaluation with interval analysis was conducted on 1st January to 31st December 2023 at the Jessop Wing Colposcopy Service, Sheffield, UK. All patients with moderate dyskaryosis or worse on referral cytology, CIN2 or worse on biopsy, were offered an HIV test.

**Results:**

Of 367 eligible patients, six (1.6%) were known HIV positive. HIV prevalence rates in patients referred with moderate or worse cytology (16.34 per 1000) (95% confidence interval 6–35), and CIN2 or worse (12.46 per 1000) (95% confidence interval 3–28) were high in comparison with overall prevalence rates for Sheffield (2.14 per 1000). HIV testing was offered to 296 (82%) patients and accepted by 246 (83%). All HIV test results were reported as not detected. Sixty‐five (18%) were not offered a test. Consultant colposcopists (*p* < 0.0001) were less likely to offer an HIV test as compared to colposcopy nurses.

**Conclusion:**

Acceptance of HIV testing in patients attending colposcopy is high, but willingness to offer the test varies at both local and national levels. Whilst there were no new HIV diagnoses in our sampled cohort, knowledge of HIV status is a key prognostic indicator and crucial for determining ongoing management. National societies must join forces and provide national guidance for the colposcopy community regarding HIV testing in colposcopy services.

**Audit registration number:** STH 12112

## 1. Introduction

Human immunodeficiency virus (HIV) testing for patients diagnosed with cervical intraepithelial neoplasia (CIN) grade 2 or worse was introduced as a standard policy in the Jessop Wing Coloscopy Unit, Sheffield, UK, on 1st January 2023. HIV testing in all patients diagnosed with cervical cancer was previously introduced in May 2021 and implemented across all hospital trusts working within South Yorkshire and Bassetlaw, working within the North Derbyshire Cancer Alliance. HIV testing was introduced for all patients diagnosed with cervical cancer as HIV testing is recommended in all AIDS‐defining conditions, which includes cervical cancer. The rationale for testing all patients with CIN2 or worse falls in line with the following: (i) recommendations to perform HIV testing in indicator conditions (any medical condition associated with an undiagnosed HIV seroprevalence ≥ 1 per 1000), including CIN [1]; (ii) 2008 National HIV testing guidelines produced by the British Association of Sexual Health and HIV (BASH), British HIV Association (BHIVA) and British Infection Society recommending HIV testing for patients diagnosed with cervical CIN2 or above [1] and (iii) the 2019 UK government’s commitment to the elimination of HIV transmission by 2030 [2].

Current departmental policy in the Jessop Wing Colposcopy Unit regarding HIV testing includes offering HIV testing to all patients referred to colposcopy services with an abnormal cervical sample of moderate or worse cytology, high‐risk human papillomavirus (hr‐HPV) detected and offering HIV testing to all patients returning for treatment for biopsy detected cervical cancer, high‐grade CIN (CIN2 or 3) or cervical glandular intraepithelial neoplasia (CGIN) of any grade. If the patient has been tested as part of antenatal care screening within the prior 12 months, with HIV not detected, then a repeat HIV test is not required (unless the patient declares a new sexual relationship or has other high‐risk authoring factors). Patients already diagnosed with HIV also do not require repeat HIV testing in the colposcopy department.

The prevalence rate for people (aged 15–59) living in Sheffield with diagnosed HIV in 2022 was 2.14 per 1000 with 95% of patients on treatment and 98% of those treated being virally suppressed. [3] The prevalence of HIV in our community of patients with moderate or worse dyskaryosis or CIN2 or worse is presently unknown.

Following implementation of HIV testing for patients diagnosed with moderate or worse referral cytology and CIN2 or worse on histology, a plan was made to audit the number of patients: (i) referred with moderate or worse dyskaryosis, hrHPV detected, or biopsy‐proven CIN2 or worse; (ii) referred with moderate or worse dyskaryosis, hrHPV detected or biopsy proven CIN2 or worse with a known diagnosis of HIV prior to referral; (iii) offered an HIV test and their acceptance of this and (iv) with HIV detected following referral to colposcopy. We consider the first year following policy implementation from 1st January 2023 to 31st December 2023.

## 2. Materials and Methods

A retrospective service evaluation was conducted between 1st January and 31st December 2023 at the Jessop Wing Colposcopy Service, Sheffield, UK. The Jessop Wing Colposcopy database was searched for all patients referred with moderate or worse dyskaryosis on cytology, and those diagnosed with biopsy‐proven CIN2 or worse. The database was cross‐referenced with the trust‐based integrated clinical environment (ICE) system (established web‐based requesting and reporting service), the Trusts histopathology laboratory reporting system and the Gateshead Cervical Screening referral service (laboratory referring abnormal cervical screening results).

Data were collated regarding date of diagnosis, age at diagnosis, ethnicity, postcode, histological diagnosis, HIV testing status and HIV test results. Patients were categorised into known HIV positive, HIV test offered and accepted, HIV test offered and declined and HIV test not offered. Reasons for declining a HIV test were recorded where an explanation was offered.

Postcodes were utilised to calculate index of multiple deprivation (IMD) quintile data using the English indices of deprivation 2019 postcode look‐up (Quintile 1 most deprived, Quintile 5 least deprived) [4]. Ethnicity was calculated using the Office for National Statistics 2021 census data, list of ethnic groups [5].

HIV testing was performed using serum samples upon the Roche Cobas Elecsys HIV combi‐PT qualitative, serologic sandwich immunoassay, which detects the HIV‐1 p24 antigen and antibodies to HIV‐1 and HIV‐2. Positive results were confirmed using the DiaSorin LIAISON XL murex HIV chemiluminescent Antigen/Antibody immunoassay. A third‐line assay, the Bio‐Rad Genscreen enzyme immunoassay was used for discrepant results.

Following cleaning and coding of data, baseline statistical analyses were performed and, where appropriate, results were analysed using chi‐squared test (“GraphPad” software).

As the study was performed in the context of a service evaluation, ethical approval was not required.

## 3. Results

Between 1st January and 31st December 2023, 372 patients referred to the Jessop Wing Colposcopy Service were identified as eligible for HIV testing, five of whom failed to attend their appointment. Of the 367 patients attending, median age was 34 years (range 23–68 years). Most were of White British ethnicity (*n* = 251, 68%), residing in areas of higher social deprivation (n‐287, 78%) (Table 1).

**TABLE 1:** Patient demographic data and HIV testing at the Jessop Wing Colposcopy Department: 1st January 2023 to 31st December 2023.

		***n***	**%**	**Known HIV**	**HIV test offered**	**HIV test offered and accepted**	**HIV test offered and declined**	**HIV test not offered**
***n***		**367**		**6**	**296**	**246**	**50**	**65**

Age at appointment (years)	≤ 25	32	9%	0	25 (78%)	22 (69%)	3 (9%)	7 (22%)
	26–35	185	50%	0	151 (82%)	117 (63%)	34 (18.5%)	34 (18.5%)
	36–50	117	32%	5 (4%)	97 (86%)	88 (79%)	9 (8%)	15 (13%)
	51–65	31	8%	1 (3%)	23 (77%)	19 (63%)	4 (13%)	7 (23%)
	> 65	2	< 1%	0	0	0	0	2 (100%)

Ethnicity	White British	251	68%	0	204 (81%)	167 (66%)	37 (15%)	47 (19%)
	White other	28	8%	0	24 (86%)	22 (79%)	2 (7%)	4 (14%)
	Asian or any other Asian background	16	4%	0	13 (81%)	11 (69%)	2 (12%)	3 (19%)
	Black or any other Black background	22	6%	4 (18%)	13 (72%)	11 (61%)	2 (11%)	5 (28%)
	Mixed or any other mixed background	15	4%	0	10 (67%)	9 (60%)	1 (7%)	5 (33%)
	Any other ethnic group	11	3%	0	11 (100%)	9 (82%)	2 (18%)	0
	Unknown	24	7%	2 (8%)	21 (95%)	17 (77%)	4 (18%)	1 (5%)

IMD quintile	1st	159		3	130	110	20	26
	2nd	56		0	43	42	1	13
	3rd	72		2	63	46	17	7
	Lower IMD quintiles	287	78%	5 (2%)	236 (82%)	198 (69%)	38 (13%)	46 (16%)
	4th	38		1	27	21	6	10
	5th	37		0	29	24	5	8
	Upper IMD quintiles	75	20%	1 (1%)	56 (75%)	45 (60%)	11 (15%)	18 (24%)
	Unknown	5	2%	0	4	3	1	1

Most were referred following a cervical sample reporting high‐grade dyskaryosis (*n* = 287, 78%) (Table 2), and the majority were seen by a Clinical Nurse Specialist (CNS) in colposcopy (*n* = 347, 95%).

**TABLE 2:** Referral criteria, histological diagnosis and HIV testing at the Jessop Wing Colposcopy Department: 1st January 2023 to 31st December 2023.

		***n***	**Percent**	**Known HIV**	**HIV test offered and accepted**	**HIV test offered and declined**	**HIV test not offered**
***n***		**367**		**6**	**246**	**50**	**65**

Referral cytology	Negative hrHPV detected	9	3%	0	6	0	3
	Borderline	41	11%	0	24	6	11
	Low grade	29	8%	0	21	5	3
	High‐grade (moderate)	188	51%	0	125	29	34
	High‐grade (severe)	80	22%	4	56	9	11
	High‐grade invasion	12	3%	2	9	0	1
	Glandular dyskaryosis	8	2%	0	5	1	2

Reviewing colposcopist	CNS	347	95%	6	239 (69%)	49 (14%)	53 (15%)
	Consultant	18	5%	0	6 (33%)	1 (6%)	11 (61%)
	Trainee colposcopist with CNS	2	< 1%	0	1 (50%)	0	1 (50%)

Final histological diagnosis	No CIN	22	6%	1	10	3	8
	CIN1	22	6%	0	12	0	10
	CIN2	162	44%	1	115	23	23
	CIN3	140	38%	3	95	22	20
	CGIN	5	1%	0	2	1	2
	CIN and CGIN	6	2%	0	6	0	0
	Cancer	8	2%	0	6	1^∗^	1^∗^

^∗^Note: All patients diagnosed with cancer offered an HIV test during their treatment if not already performed in colposcopy.

Six patients (1.6%) were known to be HIV positive and under treatment so further testing was not performed. In total, 296 (82%) were offered an HIV test, 246 (83%) accepted the offer and 50 (17%) declined the offer. A total of 65 patients (18%) were not offered an HIV test.

All patients who underwent an HIV test were HIV negative. The most common reason given for declining an HIV test was a prior HIV test (*n* = 26, 52%), followed by needle phobia (*n* = 12, 24%). Five patients (10%) regarded themselves as low risk for HIV, and in seven (14%), no reason for declining testing was provided (Table 3).

**TABLE 3:** Patient reasons for declining an HIV test.

Reason provided	*n*	Prior HIV test within 12 months	Prior HIV test within ≤ 3 years	Prior HIV test > 3 years	No documented evidence of a prior HIV test
Prior HIV test	26	10	9	4	3
Regard themselves as low‐risk	5	0	0	1	4
Needle phobia/declines blood sampling	12	0	1	0	11
No reason provided	7	0	1	1	5
Total	50	10	11	6	23

HIV prevalence rates in patients referred with moderate or worse cytology (16.34 per 1000) (95% confidence interval 6–35), and CIN2 or worse (12.46 per 1000) (95% confidence interval 3–28), were high in comparison with overall prevalence rates for Sheffield (2.14 per 1000).

Compared to all other participants (*n* = 361), patients known to be HIV positive on referral were significantly more likely to be aged > 36 years (*p* = 0.0044), of Black or any other Black ethnic background or of unknown ethnicity (*p* < 0.0001) and referred with high‐grade (severe) or severe invasive cytology (*p* = 0.0002).

Whilst on appearance patients seemed less likely to be offered an HIV test when > 65 years, this failed to achieve statistical significance and may be related to low numbers (*n* = 2) in this age group.

Ethnicity was not associated with the offer of an HIV test by colposcopy staff nor was it associated with the decline of an offer by the patient. IMD was not associated with either an offer, acceptance or decline of an HIV test.

An HIV test was significantly less likely to be offered if the patient was seen by a Consultant colposcopist (*p* < 0.0001) as compared to a colposcopy CNS.

## 4. Discussion

Despite prior guidance, there is a debate about the value of HIV screening in colposcopy services with no current national guidance provided by the NHS Cervical Screening Programme (NHSCSP) nor recommendations made by the British Society for Colposcopy and Cervical Pathology (BSCCP). In view of this lack of guidance, few colposcopy services routinely offer HIV testing to patients.

Recommendations are to test all patients with CIN2 or worse [1]. Notably, the majority with cervical dyskaryosis will not have HIV, but those who are HIV positive could be diagnosed and offered life‐saving treatment early also resulting in a reduction in transmission rates. Further benefits of diagnosing HIV in the colposcopy community are related to evidence that HIV reduces the effectiveness of the standard treatment for CIN 2/3 and cervical cancer. There are known differences in the prevalence, incidence, progression and regression of cervical lesions in females living with HIV, with the risk and severity of the lesions correlating with the degree of immunodeficiency. Furthermore, knowledge of a patient’s HIV status may influence management of CIN, as evidence suggests that standard excisional treatments for CIN are associated with higher rates of recurrence in HIV‐positive women [6–9]. For example, NHSCSP guidelines recommend that lesions less than CIN2 should not be treated, as these will often clear spontaneously and display a poor treatment response. High grade CIN in HIV‐positive women should, however, be managed according to national guidance [10].

Recurrence rates of CIN after excision with negative margins have been estimated at 20%–75% compared with 8.5%–16% in those not known to be HIV‐positive [6–9]; however, the risk of recurrence may be reduced by exposure to appropriate antiretroviral therapy (ART). Females living with HIV are also more likely to have involved margins after conisation [7, 9, 11, 12].

The prevalence rate for people (aged 15–59) living in Sheffield diagnosed with HIV in 2022 was 2.14 per 1000 [3]. In our colposcopy community, however, the prevalence rate in those referred with moderate or worse dyskaryosis was much higher, with six of 367 patients referred already diagnosed and receiving treatment for HIV. This could potentially equate to a prevalence rate of 16.34 per 1000 patients referred with cytology reporting moderate dyskaryosis or worse. Four of 321 patients diagnosed with CIN2 or worse had a known HIV diagnosis equating to a potential prevalence rate of 12.46 per 1000 patients in those diagnosed with CIN2 or worse. The cohort under study likely represents a higher risk group for HIV compared to the general population, which may explain why no new cases of HIV were identified/all had been diagnosed previously. Whilst this high risk cohort undoubtedly is a source of selection bias, both results would clearly support HIV testing within a UK‐based colposcopy service.

Data on HIV prevalence in patients attending colposcopy services in England are sparse [13]. Studies from London teaching hospitals showed an overall prevalence of 2.2%–3% in patients with cervical dysplasia of any grade [14, 15] and of 1.7% in those with high‐grade lesions.

In total, 82% of patients in this study were offered an HIV test with 83% accepting the offer, and 17% declining. Prior UK studies have reported that willingness and acceptance of HIV testing among patients was high [16, 17] with reported acceptance rates ranging between 59% and 96.5% [14, 15, 17, 18]. In the 17% declining an HIV test, 52% reported having a prior negative HIV test where 38% of these had been performed in the preceding 12 months. This falls in line with prior reported UK data, where 55% of those who declined previously had an HIV test within 1 year prior to their colposcopy appointment [14]. Up to 24% of patients declining an HIV test claimed to be either needle phobic or that they did not like injections. This is not surprising as injection or needle phobia is common and thought to affect 3.5% to 10% of the population [19]. In this situation, it would seem justified to offer an alternate option in the form of a saliva test for HIV. Prior studies have also reported that 41% of patients initially accepting HIV testing declined to wait for phlebotomy [15, 16]. This was interpreted as suggesting that point‐of‐care testing increases the coverage [15, 16], and the reason why all testing in Sheffield is performed within the realms of our colposcopy clinic.

Unfortunately, 18% of patients were not offered HIV testing and were significantly less likely to be offered HIV testing by a medical, or as in our case, Consultant colposcopist, as opposed to a colposcopy CNS. In our clinic, the CNS team have championed HIV testing within our colposcopy community, and the reluctance of medical staff is somewhat surprising, as all medical colposcopists are Gynaecological Oncologists who already routinely offer HIV testing to all patients diagnosed with cervical cancer. Potential explanations for this disparity may include differences in workflow, time pressures and training; however, further educational work clearly needs to be performed in this group of colposcopists. In general, clinician‐related barriers to the uptake of HIV testing are known to be of significance [17], with unfamiliarity with HIV testing discussions and time constraints known to be significant barriers [20]. It is also disappointing that 28% of our patients of Black or any other Black background were also not offered HIV testing as, by the ethnic group, Black Africans are more likely to be diagnosed later than the White population (59% and 43%, respectively), and HIV testing is nationally recommended in all of Black African ethnicity [20]. This significant health inequality arises from a combination of systemic, interpersonal and cultural factors yet must be tackled to break down clinician‐driven disparities in patient’s access to vital testing and care.

This study has shown that HIV testing in colposcopy services is not only acceptable to those offered but also feasible. Importantly, it has highlighted higher prevalence rates in our colposcopy community than we expected, emphasising the validity of HIV testing to both our colposcopy team and NHS trust.

This study may be limited by a degree of selection bias and its small size, but this reflects, that those offered testing, were limited by current national guidelines to test only those with a diagnosis of CIN2 or worse. The authors believe, however, that there is enough evidence to warrant HIV testing of all patients referred with abnormal cervical screening and that a visit to the colposcopy service is an opportune time to perform such a test. CIN2 or worse is not the only HIV‐associated abnormality seen in a colposcopy setting [13, 21, 22]. A prior UK study [14] identified that most females living with HIV did not have high‐grade CIN suggesting that the adoption of current HIV testing guidance in colposcopy, rather than providing routine opt‐out testing for everyone, would result in missing a substantial number (three‐quarters) of HIV diagnoses [14]. The authors also agree that offering opt‐out testing to all patients referred with abnormal cervical screening results could potentially normalise testing, reduce barriers and facilitate conversations on HIV prevention [13].

## 5. Conclusion

If we are going to achieve the UK governments commitment to the elimination of HIV transmission by 2030 [2], it is imperative that we perform HIV testing for patients with indicator conditions. A colposcopy setting sees patients of a wide age range, from multiple ethnicities and backgrounds, including those who are vulnerable and those who are otherwise medically fit, who may not access healthcare services for other reasons for many years. We agree that national societies need to join forces and provide national guidance for the colposcopy community regarding HIV testing in colposcopy services [23], be that for those with CIN2 or worse, or for all women referred with cervical screening abnormalities.

## Author Contributions

Study concept and design: Julia E. Palmer and Ella Hodson. Data collection: Ella Hodson, Rachel E. Lyon and Julia E. Palmer. Analysis and interpretation of data: Victoria L. Parker, Ella Hodson, Rachel E. Lyon and Julia E. Palmer. Drafting of the manuscript: Victoria L. Parker, Ella Hodson and Julia E. Palmer. Critical revision of the manuscript for important intellectual content: Victoria L. Parker, Benjamin I. Stone, Madeleine C. Macdonald and Julia E. Palmer. Statistical analysis: Victoria L. Parker and Julia E. Palmer. Study supervision: Madeleine C. Macdonald and Julia E. Palmer.

Dr Victoria L. Parker (corresponding author) had full access to all of the data in this study and takes complete responsibility for the integrity of the data and the accuracy of the data analysis.

## Funding

No funding was received for the conduct of this study.

## Disclosure

Dr Victoria L. Parker (Corresponding author) affirms that this manuscript is an honest, accurate and transparent account of the study being reported, that no important aspects of the study have been omitted and that any discrepancies from the study as planned (and, if relevant, registered) have been explained. All authors have read and approved the final manuscript.

## Ethics Statement

As this study was performed in the context of a service review, ethical approval was not required.

## Conflicts of Interest

The authors declare no conflicts of interest.

## Data Availability

The data that support the findings of this study are available from the corresponding author upon reasonable request.
